# Supplementary material for: Characterization of dengue cases among patients with an acute illness, Central Department, Paraguay
Source: PeerJ. 2019 Oct 9;7:e7852. doi: 10.7717/peerj.7852 (PMC6790102; doi:10.7717/peerj.7852)

Characterization of dengue cases among patients with an acute illness, Central Department, Paraguay

Alejandra Rojas, Fátima Cardozo, César Cantero, Victoria Stittleburg, Sanny López, Cynthia Bernal, Francisco Giménez, Laura Mendoza, Benjamin A. Pinsky, Yvalena Guillén, Malvina Páez, Jesse J. Waggoner

Supplemental Figures

Figure S1. DENV viral load in samples with detectable DENV NS1 antigen (●) or without detectable NS1 (▲). Bars display mean ± 95% CI. P values (t test): **** = p < 0.0001

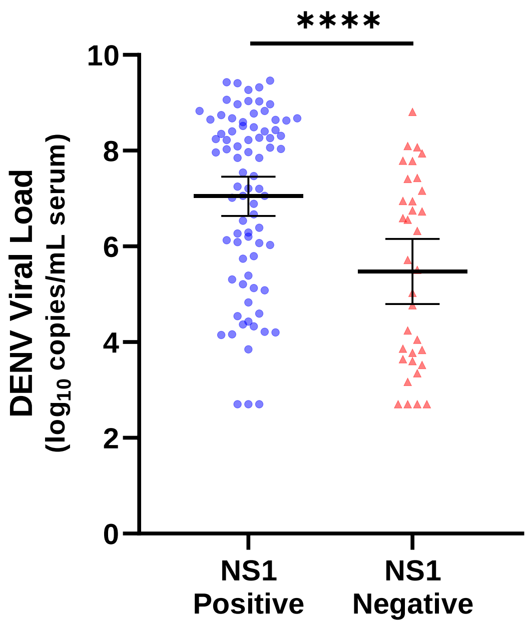

Figure S2. Distribution of DENV-positive and DENV-negative cases by epidemiologic week, 2018.

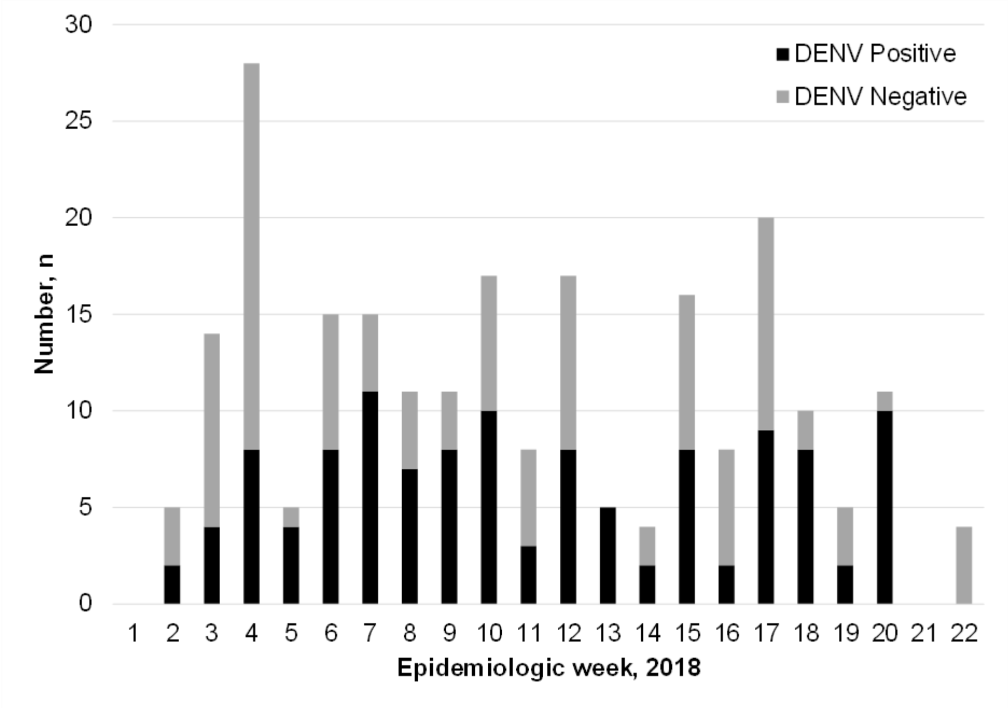

Supplement: Supplemental Information 1 [file peerj-07-7852-s002.pdf]
