# Supplementary material for: Characterization of dengue cases among patients with an acute illness, Central Department, Paraguay
Source: PeerJ. 2019 Oct 9;7:e7852. doi: 10.7717/peerj.7852 (PMC6790102; doi:10.7717/peerj.7852)
Supplement: Table S1 [file peerj-07-7852-s003.docx]

**Table S1.** Multiple linear regression (least squares model) of factors associated with DENV viral load at presentation.

| **Factor** | **Estimate** | **95% Confidence Interval** | **p-value** |
| --- | --- | --- | --- |
| Anti-DENV IgG |  |  |  |
| Negative | Reference | | |
| Positive | -1.3 | -2.1 – -0.6 | < 0.001 |
| Anti-ZIKV IgG |  |  |  |
| Negative | Reference | | |
| Positive | -0.7 | -1.48 – -0.01 | 0.047 |
| Age | -0.02 | -0.05 – -0.001 | 0.039 |
| Day of illness at presentation | -0.5 | -0.7 – -0.3 | < 0.001 |
