# Supplementary material for: Characterization of dengue cases among patients with an acute illness, Central Department, Paraguay
Source: PeerJ. 2019 Oct 9;7:e7852. doi: 10.7717/peerj.7852 (PMC6790102; doi:10.7717/peerj.7852)
Supplement: Table S2 [file peerj-07-7852-s004.docx]

**Table S2.** Association between NS1 detection among confirmed dengue cases, anti-DENV IgG and anti-ZIKV IgG.

| **Factor** | **Odds Ratio** | **95% Confidence Interval** | **p-value** |
| --- | --- | --- | --- |
| Anti-DENV IgG, negative | 1.0 | 0.3 – 3.1 | 0.928 |
| Anti-ZIKV IgG, negative | 0.1 | 0.0 – 0.3 | < 0.001 |
