# Supplementary material for: Characterization of dengue cases among patients with an acute illness, Central Department, Paraguay
Source: PeerJ. 2019 Oct 9;7:e7852. doi: 10.7717/peerj.7852 (PMC6790102; doi:10.7717/peerj.7852)
Supplement: Table S3 [file peerj-07-7852-s005.docx]

**Table S3.** Multivariable model of factors associated with hospitalization among dengue cases.

| **Factor** | **Odds Ratio** | **95% Confidence Interval** | **p-value** |
| --- | --- | --- | --- |
| Day of Illness at presentation | 1.3 | 0.9 – 1.8 | 0.117 |
| Platelet count, x 10^4^ per/µL | 0.85 | 0.76 – 0.95 | 0.005 |
